# Promoting professional identity through peer support during studies: A report on the peer support in medical education project

**DOI:** 10.3205/zma001824

**Published:** 2026-03-23

**Authors:** Iris Warnken, Ann-Kathrin Schindler, Dominik Hinzmann, Andreas Igl, Thomas Rotthoff

**Affiliations:** 1 University of Augsburg, Faculty of Medicine, Medical Didactics and Educational Research (DEMEDA), Augsburg, Germany; 2 TUM School of Medicine and Health, Department of Anesthesiology and Intensive Care Munich, Department of Clinical Medicine, Munich, Germany; 3 PSU-Akut e.V., Munich, Germany

**Keywords:** peer support, self-care, collegial care, medical studies, medical students, professional identity, medical identity, mental health

## Abstract

**Objective:**

Poor well-being, burnout and depression are prevalent among doctors, who are exposed to a variety of stresses and crisis situations. Even medical students have been described as prone to burnout due to ‘relentless demands on themselves’ [Mata DA et al., JAMA. 2015] or as vulnerable to illness from poor coping with psychosocial stress and the use of dysfunctional coping strategies [Puthran R et al., Med Educ. 2016]. In the model degree programme in human medicine at the University of Augsburg, students are offered peer support, with the aim of establishing self-care and collegial care which contribute to the development of professional identity. Collegial resources are used as both primary and secondary preventive measures in personal, study-related or clinic-related crises.

**Project description:**

Since 2022, training of student peers has been provided as part of a three-day elective course in cooperation with Munich’s PSU-Akut e.V., which, since 2019, has successfully implemented the concept of collegial support following serious incidents in the healthcare sector, for example at the University Hospital in Augsburg.

**Demand to date:**

By March 2025, 25 student peers had undergone training, which focuses on the practical and reflective practice of conversation processes, self-awareness, practising key peer communication skills, dealing with boundaries, stress-inducing thoughts, background knowledge on coping strategies and quick-acting stress-reduction strategies. As of March 2025, 42 peer-to-peer conversations had been documented. Contact was established by approaching peers in person, either by email or telephone. The main reasons for seeking support were perceived exhaustion and feeling overwhelmed by the amount of material to be covered – especially in combination with individual life circumstances, exam anxiety and perceived pressure to succeed.

**Conclusion:**

Based on initial experiences, the provision of student peer support can serve as a building block of professionally implemented identity development, in which self-care and collegial care are natural components of one’s own professional identity. The support offered by faculty as a primary and secondary preventive measure was found to be appreciated by some of the students (as expressed, for example, in evaluations and to the student and clinical mentors in mentoring groups). However, as in hospitals, such support should be continuously promoted during the establishment phase to ensure it becomes and remains widely known as a low-threshold option. Scientific proof of its effectiveness is currently being planned but is not yet available.

## Zusammenfassung

**Zielsetzung:**

Ärzt:innen sind vielfältigen Belastungen sowie krisenhaften Situationen ausgesetzt und weisen erhöhte Prävalenzen für mangelndes Wohlbefinden sowie Burnout und Depressionen auf. Bereits für Medizinstudierende werden bspw. eine Verausgabungsneigung mit „unerbittlichen Ansprüchen an sich selber“ [Mata DA et al., JAMA. 2015] oder ein ungünstiger Umgang mit psychosozialen Belastungen und dysfunktionale Bewältigungsstrategien als krankmachende Faktoren beschrieben [Puthran R et al. Med Educ. 2016] . Im Modellstudiengang Humanmedizin der Universität Augsburg erhalten Studierende das Unterstützungsangebot eines studentischen Peer Supports. Ziel ist es, Selbst- und kollegiale Fürsorge sowie die Nutzung kollegialer Ressourcen im Sinne eines primär-, aber auch sekundärpräventiven Angebots bei persönlichen als auch studiums- oder klinikbezogenen Krisen als wichtige Bestandteile einer entstehenden professionellen Identität bereits im Medizinstudium zu etablieren.

**Projektbeschreibung:**

Die Ausbildung der studentischen Peers erfolgt seit 2022 im Rahmen eines dreitägigen Wahlpflichtfaches. Kooperationspartner ist der Verein PSU-Akut e.V. aus München, der das Konzept der kollegialen Unterstützung nach schwerwiegenden Ereignissen im Gesundheitswesen seit 2019 erfolgreich bspw. im Universitätsklinikum Augsburg implementiert hat.

**Bisherige Nachfrage:**

Bis März 2025 wurden insgesamt 25 studentische Peers ausgebildet. Schwerpunkte der Ausbildung bilden das praktische und reflektierte Üben der verschiedenen Gesprächsabläufe, die Selbsterfahrung, das Einüben der Schlüsselkompetenzen von Peer-Kommunikation, Umgang mit Grenzen, stressverstärkenden Gedanken, Hintergrundwissen zu Bewältigungsstrategien sowie schnell wirksame Strategien zum Stressabbau. Bisher wurden 42 Peer-to-Peer-Gespräche dokumentiert (Stand März 2025). Die Kontaktaufnahme erfolgte über persönliche Ansprache der Peers, per E-Mail oder telefonisch. Anlässe sind v.a. wahrgenommene Erschöpfung, Überforderung mit der Stoffmenge, vor allem in Kombination mit individuellen Lebensumständen, Prüfungsängste und wahrgenommener Erfolgsdruck.

**Schlussfolgerung:**

Das Angebot eines studentischen Peer Supports kann entlang der ersten Erfahrungen einen Baustein von curricular implementierter professioneller Identitätsentwicklung darstellen, in welcher Selbst- und kollegiale Fürsorge selbstverständliche Bestandteile der eigenen professionellen Identität darstellen. Das Unterstützungsangebot als primär- wie sekundärpräventive Maßnahme der Fakultät wird von einem Teil der Studierenden anerkennend wahrgenommen (formuliert bspw. in Evaluationen sowie gegenüber den studentischen wie klinischen Mentor*innen in Mentoring Gruppen), muss allerdings – wie in den Kliniken – in der Etablierungsphase kontinuierlich beworben werden, um als niederschwellige Unterstützungsoption weiter bekannt zu werden und zu bleiben. Ein wissenschaftlicher Wirksamkeitsnachweis ist in aktueller Planung, steht zu diesem Zeitpunkt indes noch aus.

## 1. Introduction

The increased prevalence of poor well-being, burnout and depression among doctors has often been described in international reviews [1], [2], [3]. This problem is closely linked to the professional identity of medical professionals, which is shaped by working beyond individual stress limits and by exposure to crisis events [4]. Such experiences are part of everyday medical work but must be processed to reduce negative consequences for mental health, patient safety and healthcare quality [5]. 

Institutionally anchored collegial peer support can be a first step towards stabilising and supporting medical professionals after stressful incidents [6]. In the United States, support programmes for second victims – that is, medical personnel who themselves become victims because of traumatic events in the workplace [7] – have been established and evaluated. Examples include the Rise programme at Johns Hopkins University [8] and the forYou programme at the University of Missouri [9]. In Germany, PSU-Akut e.V. is a leader in introducing PSU peer networks in clinics [6] and setting up specialist coordination centres with telephone helplines [10].

Studies have indicated that medical students can develop unfavourable strategies for dealing with psychosocial stress before and during their studies [11], [12]. In addition to study-related factors, such as a high workload or demanding exam formats, these stresses can arise from social conflicts, patient crises, deaths, experiences of discrimination, sexism, financial problems and multiple burdens. They can not only lead to overload but also promote unhealthy habits – such as excessive willingness to perform or ignoring one’s own limits, as exemplified by medical role models, among others [13]. In the long term, these coping strategies can not only affect one’s own well-being and satisfaction but also impair interactions with patients [14] or influence the overall quality of care [15]. Such patterns of behaviour, whether observed or experienced first-hand, can influence the professional identities of prospective doctors through conscious and unconscious processes [16]. They are also reflected in the courses of the longitudinal *Augsburg Maturitas curriculum*, which promotes professional identity development across all semesters [https://www.uni-augsburg.de/de/fakultaet/med/profs/medizindidaktik/maturitas/]. In the Peer Support in Medical Education project, student peers are trained to act as first points of contact for medical students experiencing excessive stress. Especially in this initial phase of professional socialisation, the experience of collegial support can represent a shift in thinking about how to manage and disclose excessive stress, serious events or even one’s own illnesses – ideally in the clinical context. 

Medical students are becoming more aware of the relevance of mental health [17], and it seems that they may be willing to communicate problems openly and to seek and accept support. This can be interpreted as a positive prerequisite for reflecting on previous behaviour patterns and initiating change [18]. According to Cruess et al. [19], in the development of professional identity, the core of one’s pre-existing personal identity should not be suppressed by community norms, and faculty members must show understanding and support for competing discourses [19]. 

To address these challenges, international programmes have been developed to promote the mental health of medical students, such as mindfulness training and peer-to-peer support programmes [16], [20], [21]. These programmes aim to help students maintain their own health while protecting patient safety in clinical settings [22]. Thus far, they have not shown any link to the concept of professional identity development in medical schools. Their primary aim – in the sense of behavioural prevention – is to teach individual stress management skills, increase well-being and reduce stigma surrounding the stresses of medical school, the disclosure of mental health issues and the acceptance of professional help [23]. 

In the model degree programme in human medicine in Augsburg, we are building on existing programmes through the *peer support in medical education* project. We see the importance of primary preventive measures – such as teaching coping skills and psychoeducation – self-care and collegial care, as well as encouragement to seek support at an early stage in the event of a crisis as a secondary preventive measure [24], as part of developing the professional identity of prospective doctors. In the following, we present the *peer support in medical education *project as a form of situational prevention in medical faculties, as well as its use by students thus far. Nevertheless, fundamental, system-relevant situational prevention is still lacking, even though model study programmes such as those in Augsburg are attempting to reduce excessive stress.

## 2. Description of the peer support in medical education project

### 2.1. Clinical, collegial peer support as a starting point

The *peer support in medical education* pilot project was designed in collaboration with PSU-Akut e.V., a non-profit organisation that specialises in providing psychosocial support to nurses, doctors, medical assistants and other healthcare professionals, which is funded by the Bavarian State Ministry of Health and Care and financially supported by the Bavarian State Medical Association, among others. PSU-Akut e.V. focuses on implementing collegial peer support teams for healthcare personnel who offer short-term counselling to stabilise and relieve stress in the event of an incident. With the help of PSU-Akut e.V., peer support structures have been successfully established in numerous clinics at various care levels as well as at university hospitals [25]. Since 2020, the PSU Peer Network has also been implemented at University Hospital Augsburg, where it is being continuously expanded [10], having been made permanent by hospital management since October 2024. If necessary, peers trained by PSU-Akut e.V. refer affected persons to appropriate specialist and counselling services.

### 2.2. Peer support in medical education

In cooperation with PSU-Akut e.V., with funding from the Volkswagen Foundation (Grant 98539), the concept of peer support was adapted in 2022 at the Augsburg Faculty of Medicine to include exceptional stress – especially in medical studies – as a primary and secondary preventive measure [11]. The *peer support in medical educatio*n project aims to provide primary preventive support to students at an early stage of their professional identity development to help them develop an awareness of their own health as a prerequisite for their future careers, reflect on their tendency to overexert themselves and strengthen their resilience. Corresponding training objectives have become increasingly important in recent years and have also been anchored in the National Competence-Based Learning Objectives Catalogue for Medicine (NKLM). Learning objectives include the early recognition of signs of physical and psychological stress in oneself and others, awareness of one’s own limits of resilience, the application of individual strategies for coping with and reducing stress and the acceptance of professional help [https://nklm.de/zend/menu] as a secondary preventive measure. The existence and use of low-threshold, readily available support services provided by trained peers – who also act as mediators for professional support services, such as the Augsburg clearing consultation for medical students – can be a first step towards a culture of increased self-care and collegial care awareness during studies and in later clinical practice. The focus of (student) peer support is on a distinct form of social support characterised by a clear basic attitude (acceptance, appreciation and compassion), active listening and the provision of reassurance and information about typical experiences (psychoeducation), coping strategies and further support services [26]. 

#### 2.2.1. Training of student peers 

Since 2022, 17 medical students from Augsburg (peers) and eight students from the Technical University of Munich have been trained and professionally supported by the lead author (a PSU trainer with many years of experience in the psychosocial field who has degrees in sociology, psychology, education and physiotherapy) and have received hourly support from a subject matter expert from PSU-Akut e.V. The first two training days (7 hours each) are held consecutively, with an assignment on the topic completed independently before a seminar day, which takes place on a weekend approximately 3–4 weeks later. In their role as peers, the students receive additional professional supervision from the head psychologist at the Psychiatry and Psychotherapy Clinic at the University of Augsburg as they progress and as required. The psychiatric-psychotherapeutic clearing consultation service offered by the Chair of Psychiatry and Psychotherapy at the University of Augsburg is an important referral service and fallback option for student peers. A PSU-Akut e.V. telephone helpline is also available. 

Peer training includes scientific basics on stress, stress reactions and effects and coping with (secondary) traumatic experiences. The focus is on the practical and reflective practice of key skills in structured peer communication in line with the PSU concept, including dealing with one’s personal limits and stress-intensifying thoughts, as well as background knowledge on stress, trauma and coping strategies. It also covers various forms of conversation and dialogue following different stressful experiences as well as quick and effective strategies for stress reduction. Approximately one-third of peer training consists of exemplary case studies of serious clinical situations, and the rest consists of crisis situations during the course of studies. These crises are usually perceived as study-related challenges that are often accompanied by personal problems and can lead to intense stress and reduced cognitive performance, even to the point of feeling unable to act.

Discussions take place either formally – registered via email or telephone – or informally, through personal contact with a peer or a student. In both cases, it is essential that roles are clearly and consciously defined and that the boundaries of initial contact are observed. Unlike conversations with family or friends, peer conversations follow a clearly structured process that moves from stressful experiences in the past to the current situation as the focus, then to a future-orientated solution, with the restoration of stability and the ability to act at the forefront. In the event that those seeking advice exhibit questionable pathological symptoms, peers are trained to maintain their boundaries and to refer them to professional services, such as the Augsburg Clearing Consultation Hour. The possibility of rapid referral is essential in the context of collegial support for serious incidents and crises.

The student peer programme for the transition phase to the practical year at University Hospital Augsburg is currently being further developed. To this end, pairs of student and medical peers are formed. The aim is to create a collegial support structure for the transition from studies to professional life. 

#### 2.2.2. Integration of peer support into the curriculum for professional identity development

From the beginning of their studies, medical students in the Augsburg model programme are made aware of the importance of the reflective development of a professional identity. The *peer support in medical education *project complements the following:

1. The longitudinal *Maturitas* curriculum, which includes courses on topics such as reflection on personal experiences during studies, self-perception and the development of one’s own identity and medical role, as well as constructive ways of dealing with challenges.
2. The *Maturitas* mentoring programme, which initially involves students from higher semesters. From the fifth semester onwards, experienced doctors take on the role of mentors.

Both programmes provide space and a framework for reflective engagement with one’s own development through the help of role models. Peer support was implemented as the third pillar of the programme for professional identity development, with the aim of bringing about a cultural change in dealing with challenges and serious stress in studies and clinical practice. Self-care and collegial care can open up a low-threshold path to accepting professional help – without damaging the professional, resilient self-image of supposedly invulnerable doctors. Rather, self-care and collegial care both complement this with a further dimension of personal responsibility: the ability to recognise one’s own need for help and to accept support.

Students are introduced to the possibility and use of peer support from *Ersti-Woche* (freshers’ week) – an introductory week for first-semester students organised by the student council – and are repeatedly made aware of this support option through *Maturitas *courses and mentoring.

#### 2.2.3. Communication of the project 

In the two years since the implementation of peer support, considerable efforts have been made to raise awareness of the project among students. First, the project is mentioned and explained in the *Maturitas* introductory lecture. Posters with a specially designed key visual have been put up in several locations in the faculty, and a dedicated website with information and photos of the first peers has been created. All mentors and lecturers with relevant topics are informed of the project.

Since the second year of the project, new students have received various items of merchandise featuring a QR code linking to the website. Trained peers have been given their own peer support T-shirts to make them more visible during studies. Before semester exams, first-semester students attend small events linked to seminars, in which they receive tips on stress management and information about this collegial, non-clinical support service, as well as details of peer consultation hours during exam periods. 

At the beginning of the third year, special flyers and pens (with QR codes), as well as new, attractive merchandising products, are distributed. In addition, a peer support Instagram account [https://www.instagram.com/peersupport_med_uniaux/] has been well received. This is intended to further increase visibility and disseminate target group – specific information, such as tips, strategies or information about the consequences of suppressed stress. Initial contact is also offered via the platform.

Through this targeted and repeated promotion of *peer support in medical education*, students are informed that trained student peers are available as low-threshold discussion partners for mental health issues during their studies. These peers can be contacted for personal problems as well as for stress related to studies and clinical work. As trained guides, they can refer students to additional support services. The low-threshold, collegial support provided by student peers is intended to reduce stress, restore the ability to act and provide access to existing individual and/or new coping resources. It is important to recognise that most stress reactions form an appropriate response to abnormal events and that appropriate self-care – referred to in the clinical setting as staff safety – has a positive effect on patient safety [22].

## 3. Previous use of peer support

Conversations take place in a structured manner, in compliance with defined quality standards (guidelines for psychosocial emergency care – PSNV [27]), and in a protected, confidential setting. The individually perceived severity of an event determines the course of the conversation (PSU-Akut e.V. concept). Peers are trained to empathise with stressful situations, listen actively without pathologising stress reactions and recognise when the person they are supporting should be referred to professional standard care structures. If necessary, students with mental stress or illness can contact the clearing consultation service implemented at the faculty of medicine and offered by the chair of psychiatry and psychotherapy at any time – together with the peer, if desired. Thus far, 31 formal consultations (registered by those seeking advice) and 11 informal ad hoc consultations (with personal contact possible on both sides) have been held. Almost all of those seeking advice have been women, with female students accounting for between 59 and 67 per cent of the total. Seventeen students have been referred to specialist services.

The reasons for seeking advice vary, and the focus is currently on the following:

- Overwork combined with individual life circumstances, especially multiple stresses with pronounced exhaustion.
- Procrastination.
- Depressive symptoms and/or signs of burnout.
- Private separations.
- Anxiety, especially exam anxiety.
- Loneliness, including social withdrawal – for example, due to a high commitment to learning.
- Effects resulting from the stressful experiences described, such as increasing learning deficits, learning blocks, loss of motivation or failure to pass exams.

Regular requests for meetings have been received – albeit hesitantly – particularly during the first weeks of medical studies. In most cases, one meeting is sufficient to restore the student’s confidence and help them regain self-efficacy. However the option of a second conversation is rarely taken up immediately but is instead kept as a “backup”, according to the students themselves, in case of a another deterioration in mental well-being. The duration of conversations varies between 20 and 90 minutes. Contact with peers is established via a dedicated email address, by telephone or in person. Initial feedback is provided within 24 hours, and a meeting is offered within 48 hours of contact (see table 1 (Tab. 1) and table 2 (Tab. 2)). 

At the beginning of the project, there was uncertainty about whether peers would maintain confidentiality, as most students knew each other. In addition, medical mentors were told that the perceived burden of contacting peers was too low, while students expressed a desire to seek support for more serious problems. Some students stated that they had endured what they perceived as excessive stress for too long – an experience similarly described among doctors in clinical practice [28], [29].

Of the 17 trained peers in Augsburg, eight are currently (March 2025) available for conversations, with some having left the faculty for their practical year. In addition to offering voluntary conversations, two trained peers are responsible for the Instagram account [https://www.instagram.com/peersupport_med_uniaux/], which was established in autumn 2024. A three-day seminar is mandatory for those wishing to work as peers, but not every participant goes on to take an active peer role. When asked, the eight active peers said they had felt well prepared for their role thanks to the training. Regular refresher courses to ensure competence have been requested and take place on a regular basis. Supervision is offered once a semester and as needed, although this has not been necessary thus far. To date, there have been no problematic conversations or cases of peers being overwhelmed. They have various options to fall back on, including the university’s clearing house and the PSU-Akut e.V. helpline, which guarantees immediate professional advice and support in acute situations. 

To expand the *peer support in medical education* project, students at the Technical University of Munich were trained for the first time in the summer semester of 2024. The project is to be implemented here from the summer semester of 2025, as soon as the basic structures for establishing and supporting peers have been created. Other medical faculties have also expressed interest. 

## 4. Discussion

The *peer support in medical education* project, an institutionalised, collegial support system, addresses the strengthening of self-care and collegial care as part of professional medical identity. Awareness of these aspects is already required for medical training, in accordance with NKLM [https://nklm.de/zend/menu] and the Geneva Declaration [30]. Recognising signs of one’s own stress and applying appropriate coping strategies are defined as learning objectives ([https://nklm.de/zend/menu], see chapter VIII.6-03). This project offers an approach to providing training and raising awareness of the use of psychosocial support structures during medical studies [15]. The importance of self-care and collegial care for one’s own professional identity is addressed and made tangible through a concrete offering. Following on from this, ongoing discussion is offered – in the sense of reflection on whether or not to take up the support – in both *Maturitas* courses and mentoring.

An important aspect of this project is the introduction of low-threshold access to peer support, which enables students to seek help at an early stage, before stressful experiences develop into more serious mental health problems. An initial evaluation of the use of *peer support in medical education* showed that it is generally accepted for various counselling needs. Data on the development of medical students’ mental health during their studies suggest that demand for peer support is still lower than the potential need [31]. Thus far, the majority of requests for peer support have come from first-semester students, which could indicate challenges in their transition to university [11]. Particularly in the mentoring groups, adaptation problems are discussed, for example, in relation to excessive demands arising from running one’s own household, the loss of familiar surroundings – such as family, friends, hobbies and previous structures – as well as adapting to a new environment at university or medical school. This is compounded with unfamiliar learning volumes, exam formats and over all the demands of a new role. 

Possible inhibitions may include concerns about fellow students maintaining confidentiality or the perceived lack of relevance of perceived stress. This hesitation in seeking help – sometimes lasting until stress reactions are no longer compatible with everyday life – could resemble behavioural patterns that have been described among doctors [32] and are thus exemplified by students. These patterns are often expected, whether consciously or unconsciously [12]. These parallels highlight the need to promote a culture of self-care and collegial care in the training of medical students, coupled with openness and the acceptance of services offering professional help. However, according to informal statements by some students, the mere existence of a support service addressing the importance of self-care seems to have had a primary preventive effect. 

The further development of *peer support in medical education* through student events, the Instagram account [https://www.instagram.com/peersupport_med_uniaux/], a new podcast [https://open.spotify.com/show/45YHcT3gwWdJksO1pZEtNg] and other components of the *Maturitas* programme aims to reduce potential inhibitions by taking the following actions:

- Addressing the relevance of one’s own health – not just reflecting on emotionally sensitive forms of communication with patients [33] as a prerequisite for professional practice.
- Increasing awareness of how to deal with stress – without questioning the course itself and/or the future profession or minimising the enjoyment of the work.
- Continuing a target group-oriented communication strategy.
- Cross-locating training and consultation opportunities with student peers.

The peer support service operates at the intersection of the need to expand individual stress limits for clinical medical work and the simultaneous need to maintain the limits necessary for one’s own health. One question that remains open concerns the demand for counselling after serious incidents in clinical practice – such as traineeships or internships – which has thus far hardly been utilised. Although this has been discussed in informal conversations among lecturers, mentors or the student council, the true extent of the demand remains unclear. 

Despite the Peer Support in Medical Education project’s positive beginning, the question of its long-term effectiveness and acceptance remains open. A systematic, evidence-based study of the effects of the programme on the mental health and professional identity development of medical students seeking support is currently being planned. The literature describes a positive effect on trained peers’ compassion and increased self-efficacy [23]. A qualitative long-term study on the effects of peer training and, in particular, peer activity on one’s own professional identity is currently in preparation. However, the question of whether it is possible in the early stages of professional identity development – during university studies – to correct the widespread image of healthy, always available and invulnerable doctors through preventive measures such as peer support must remain unanswered at this point.

## 5. Conclusion

*Peer support in medical educatio*n makes it possible to integrate psychosocial support into medical training and to promote the development of a healthy professional identity. This collegial support service is not intended to replace clinical support services but rather to serve as an interface and generate a supportive learning atmosphere. The findings thus far underscore the importance of such services, while also highlighting the challenges involved in their acceptance and use – especially in the context of unconsciously conveyed role models in the hidden curriculum. For a pilot project that is still being developed and adapted, it is not yet possible to assess the long-term effects on students seeking advice or on their peers, but insights, initial findings and ideas for knowledge-driven research projects can be identified.

## Notes

### Authors’ ORCIDs

- Iris Warnken: [0009-0000-8497-2541]
- Ann-Kathrin Schindler: [0000-0002-2293-2357]
- Dominik Hinzmann: [0000-0001-5943-352X]
- Thomas Rotthoff: [0000-0002-5171-5941]

### Funding

The project was funded by the Volkswagen Foundation, Grant 98539.

## Competing interests

The authors declare that they have no competing interests.

## Figures and Tables

**Table 1:**
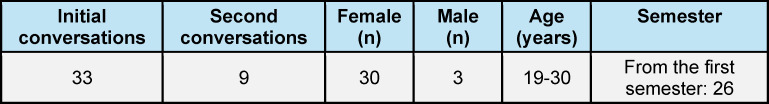
Progress between October 2022 and March 2025

**Table 2:**
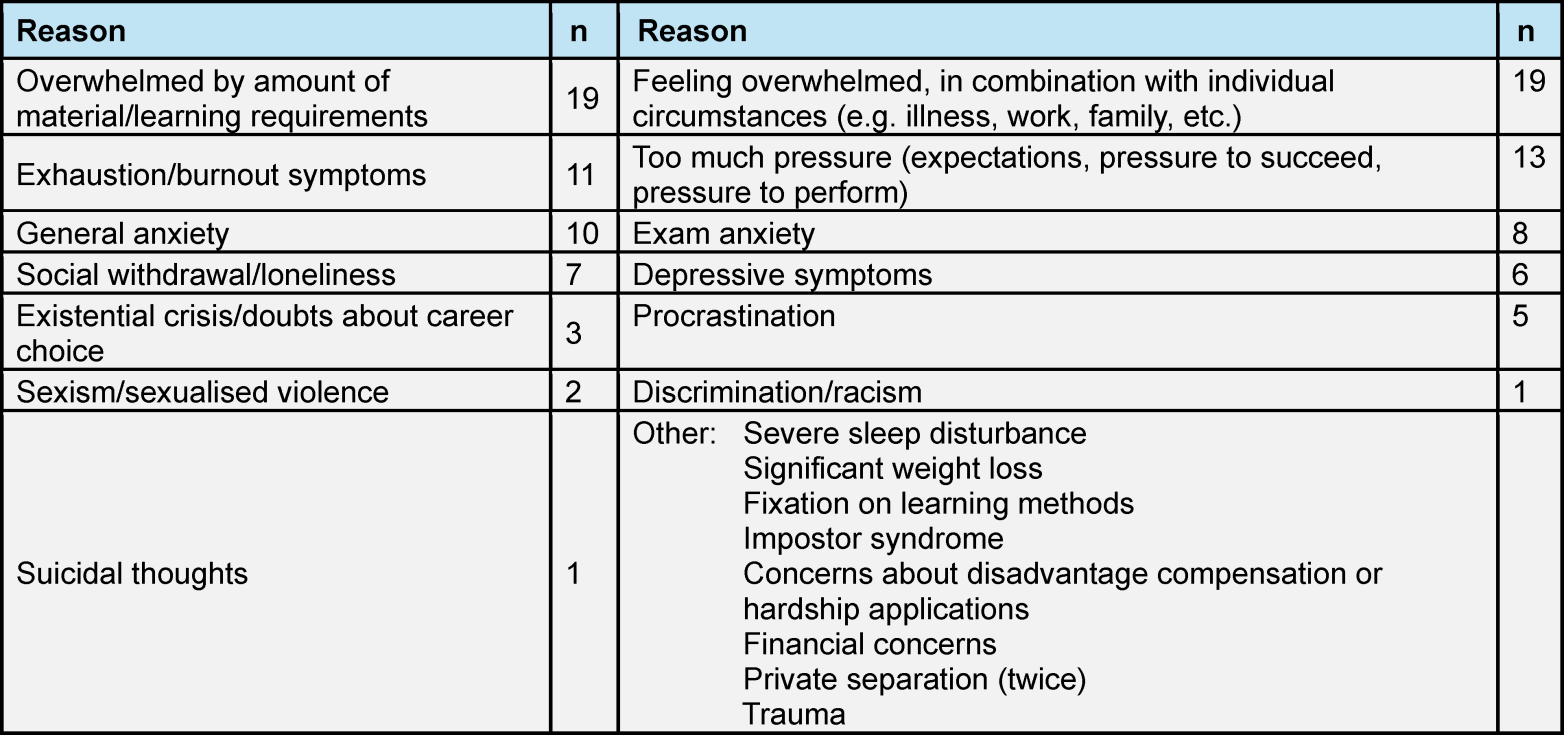
Reasons for counselling
